# Nanomedicine-Mediated Therapies to Target Breast Cancer Stem Cells

**DOI:** 10.3389/fphar.2016.00313

**Published:** 2016-09-13

**Authors:** Lili He, Jian Gu, Lee Y. Lim, Zhi-xiang Yuan, Jingxin Mo

**Affiliations:** ^1^College of Pharmacy, Southwest University for NationalitiesChengdu, China; ^2^Pharmacy, School of Medicine and Pharmacology, The University of Western Australia, CrawleyWA, Australia; ^3^Department of Pharmacy, College of Veterinary Medicine, Sichuan Agricultural UniversityChengdu, China; ^4^Key Laboratory for Stem Cells and Tissue Engineering (Sun Yat-sen University), Ministry of EducationGuangzhou, China

**Keywords:** nanomedicine, breast cancer, breast cancer stem cells, drug delivery, targeted therapy

## Abstract

Accumulating evidences have suggested the existence of breast cancer stem cells (BCSCs), which possess the potential of both self-renewal and differentiation. The origin of BCSCs might have relationship to the development of normal mammary stem cells. BCSCs are believed to play a key role in the initiation, recurrence and chemo-/radiotherapy resistances of breast cancer. Therefore, elimination of BCSCs is crucial for breast cancer therapy. However, conventional chemo and radiation therapies cannot eradicate BCSCs effectively. Fortunately, nanotechnology holds great potential for specific and efficient anti-BCSCs treatment. “Smart” nanocarriers can distinguish BCSCs from the other breast cancer cells and selectively deliver therapeutic agents to the BCSCs. Emerging findings suggest that BCSCs in breast cancer could be successfully inhibited and even eradicated by functionalized nanomedicines. In this review, we focus on origin of BCSCs, strategies used to target BCSCs, and summarize the nanotechnology-based delivery systems that have been applied for eliminating BCSCs in breast cancer.

## Introduction

Breast cancer is the most frequently diagnosed cancer and the leading cause of cancer death among females worldwide, accountings for 25% of all cancer cases and 15% of all cancer deaths among females (Torre et al., 2015). Accumulating evidence indicates that the local recurrent and/or distant metastatic tumors, the major causes of lethality in the clinic, are related to the aggressive phenotype of a small fraction of cancer cells loosely termed as cancer stem cells (CSCs), tumor initiating cells (TICs), or cancer metastasis-initiating cells (CMICs) (Geng et al., 2014). Breast cancer stem cells (BCSCs) were isolated and identified initially by Al-Hajj’s group (Al-Hajj et al., 2003). Since then, it has been revealed that BCSCs play a key role in not only the original tumorigenicity but also the local invasion and migration propensity of the breast cancer cells (Clarke, 2005; Moncharmont et al., 2012; Chen et al., 2013). BCSCs are shown to exhibit unique growth resilience, including the capacity for self-renewal, differentiation potential, and resistance to most anti-cancer agents including chemo and/or radiotherapy, all of which are believed to contribute to the development and overall aggressiveness of the recurrent or metastatic lesions (Geng et al., 2014). Moreover, the drug resistance of tumor seems to be correlated with some properties of CSCs, such as ability of DNA repairing and overexpression of drug efflux transporters and antiapoptotic proteins (Vinogradov and Wei, 2012). Therefore, the stem cell model of mammary tumorigenesis has important clinical implications for the treatment of breast cancer. The development of new therapeutic strategies designed to target BCSCs may ultimately result in more effective interventions for the treatment of breast cancer (Dontu et al., 2003).

An increasing number of therapeutic agents that have effects on eliminating or inhibiting BCSCs have been proposed and confirmed, such as salinomycin (Muntimadugu et al., 2016), disulfiram (Yip et al., 2011), chloroquine (Liang et al., 2016), curcumin (Gülçür et al., 2013), and siRNA (Zuo et al., 2016). However, most of these agents have characteristics limiting their effective applications *in vivo*, including poor solubility, off-target effects, instability, short circulation half-life, undesirable biodistribution and low therapeutic indices (Hu et al., 2012). Therefore, the development of BCSCs-targeting drug/gene delivery systems is desirable to optimize anti-BCSCstherapies.

Nanotechnology has shown immense potential for cancer detection, prevention, and treatment. Nanomedicines have revolutionalized drug delivery, allowing for therapeutic agents to selectively targeting tumor tissue and cancer cells, while minimizing toxicity to normal cells (Gao et al., 2014). Nanocarriers are ideal platform for successful BCSCs-specific therapies because they possess the properties of high drug loading capacity, solubility enhancement effects, site-specific delivery mechanism that avoids drug deposition in normal cells and tissues, negligible release of drug prematurely, and controlled release mechanism that provides effective drug doses to the target site (Estanqueiro et al., 2015). A wide range of nanocarriers (e.g., polymeric nanoparticles, metal nanoparticles, polymeric micelles, liposomes, and carbon nanotubes) has demonstrated significant potential in CSCs-targeted delivery (Shen et al., 2016). However, the application of nanotechnology specifically to develop BCSCs-targeted therapies is still fraught with challenges and barriers, with many issues remaining unresolved. In this review, we briefly discuss the origin and properties of BCSCs, and provide a summary of the latest developments in nanomedicine approaches for BCSCs-targeted therapy in recent literature.

## BCSCs Theory

### Mammary Stem Cells and Origin of BCSCs

The mammary gland is a unique organ that undergoes extensive remodeling and differentiation even in adults (Polyak, 2007). It exhibits unique developmental features during puberty, pregnancy and lactation. For each round of pregnancy, the mammary gland undergoes sequential cycles of proliferation, differentiation and apoptosis, while alveolar ducts form and grow, differentiate to produce milk and, after lactation, cease, revert and regress to the pre-pregnancy state (Williams et al., 2009). All of these suggest the presence of mammary stem cells as the basis for the capacity of renewal (Williams et al., 2009). The existence of stem-like cells in normal breast tissue has been proven by several studies (Dontu et al., 2003, 2004; Liu et al., 2005). These cells are undifferentiated cells, which posses the ability of self-renewal, and can differentiate into alveolar epithelial cells, ductal epithelial cells, and myoepithelial cells (Wicha et al., 2003). Mammary stem cells also demonstrated a significant genes expression overlap with embryonic, neuronal, and hematopoietic stem cells (Wicha et al., 2003).

**FIGURE 1:**
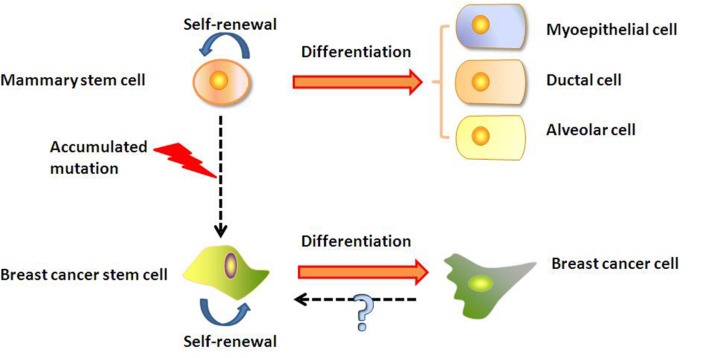
**Mammary stem cells and origin of BCSCs.** BCSCs may be derived from transformation of mammary stem cell. Alternatively, BCSCs may arise from differentiated cancer cells through an unknown mechanism.

The origin of BCSCs is still being debated (**Figure 1**). A hypothesis that has gained considerable interests in recent years postulates that the BCSCs may be derived from normal mammary stem cells transformed by the deregulation of normal self-renewal (Dontu et al., 2003, 2004). It was found that normal mammary stem cells and BCSCs share many characteristics, such as telomerase expression, self-renewal ability, differentiation capability, apoptosis resistance, as well as ability to home to specific sites (Dontu et al., 2003; Wicha et al., 2003). Stem cells have the machinery to activate self-renewal and therefore may require fewer mutations to maintain it than more differentiated cells would need to activate it ectopically (Passegué et al., 2003). In addition, mammary stem cells persist much longer and therefore have much greater opportunities to accumulate mutations than more differentiated cells. Those accumulated mutations may cause the normal stem cell to gradually change into BCSCs. Another hypothesis suggests that BCSCs may be derived from differentiated cells (Lu et al., 2016), but the mechanism is still uncertain. Guo et al. (2012) found that transient coexpression of exogenous Slug and Sox9 sufficed to convert differentiated luminal cells into mammary stem cells with long-term mammary gland-reconstituting ability, which also promoted the tumorigenic and metastasis-seeding abilities of human breast cancer cells. This hypothesis was also supported by Koren et al. (2015) findings that the expression of PIK3CAH1047R in lineage-committed basal Lgr5-positive and luminal keratin-8-positive cells of the adult mouse mammary gland evoked cells to dedifferentiate into a multipotent stem-like state.

### Microenvironment of BCSCs

Just as normal stem cells are regulated by their microenvironment, or niche, BCSCs interact with and are regulated by the factors in their microenvironment. These factors include fibroblast stimuli, immune cells, autocrine signals, and extracellular matrix (ECM) components, as well as physical/chemical factors such as oxygen pressure, nutrients levels, and pH (Bozorgi et al., 2015). Growth factors and cytokines released by tumor cells and cancer-associated fibroblasts and immune cells have strong effects on the survival and metastasis of BCSCs (Korkaya et al., 2011; Bozorgi et al., 2015; Brooks and Wicha, 2015). Korkaya et al. (2012) found the activation of an interleukin-6(IL-6) inflammatory loop mediated trastuzumab resistance in HER2^+^ breast cancer by expanding the cancer stem cell population. Ginestier et al. (2010) revealed that CXCR1 blockade selectively targeted human BCSCs and prevented tumor formation *in vitro* and in xenografts. Moreover, chronic exposure of epithelial cells to high levels of bone morphogenetic protein 2 (BMP2) has recently been demonstrated to initiate stem cell transformation toward a luminal tumor-like phenotype (Chapellier and Maguer-Satta, 2016). Carcinogen-driven deregulation of the stem cell niche could therefore represent a driving force to promote transformation and dictate the ultimate breast tumor subtype (Chapellier and Maguer-Satta, 2016), which in turn suggests that the BCSCs niche is a potential target for anticancer therapy. This strategy has yet to be sufficiently explored (LaBarge, 2010).

### Phenotyping of BCSCs and Marker

The first report of isolation and identification of BCSCs was by Al-Hajj et al. (2003), who designated them as CD44^+^CD24^-/low^ lineage^-^. When xenotransplanted into mice, 1000s of these cells were enough for the initiation of tumors, while for the unsorted population, about 50,000 cells were needed (Carrasco et al., 2014). CD44^+^/CD24^-/low^ cells have evident stem cell features. Ponti et al. (2005) isolated and propagated BCSCs from breast carcinoma cell line and breast cancer lesions. The cultured cells were named CD44^+^/CD24^-^ and Cx43^-^, and found to overexpress the neoangiogenic and cytoprotective factors, the putative stem cell marker Oct-4, and gave rise to new tumors with as few as 10^3^ cells injected into the mammary fat pad of SCID mice. The CD44 was positively associated with stem cell-like characteristics and the CD24 expression was related to differentiate epithelial features (Park et al., 2010).

Expression of CD133 (Prominin-1), which is a 120 kDa glycoprotein that localizes to plasma membrane (Mizrak et al., 2008), is used as a marker to identify TICs or BCSCs in breast tumors (Meyer et al., 2010). CD133^+^ tumor cells could form complete tumors, and CD133 expression was proved to be closely related to tumor size, recurrence, metastasis, clinical stage and overall survival in breast cancer patients (Zhao et al., 2011; Aomatsu et al., 2012). Also, *in vitro* and xenotransplantation assays revealed that CD133^+^ cancer cells have enhanced tumor initiating ability and drug resistant phenotype (Zobalova et al., 2008; Mine et al., 2009; Wang et al., 2010; Swaminathan et al., 2013).

Aldehyde dehydrogenase (ALDH) has been described as a marker of both normal and malignant breast stem/progenitor cells (Ginestier et al., 2007; Ricardo et al., 2011). ALDH converts retinol to retinoic acid, and is a putative enzyme having important properties in differentiation pathways in normal as well as cancer stem cells (Lohberger et al., 2012; Kesharwani et al., 2015). ALDH overexpression has been correlated with increased tumorigenesis in comparison to CD 44^+^ cells alone, indicating ALDH as a specific marker of BCSCs in breast cancers (Vira et al., 2012). ALDH1A1 is an isoform of ALDH used in targeting BCSC and it has been found to be responsible for chemo- and radiotherapy-resistance (Keysar and Jimeno, 2010; Subramaniam et al., 2010; Croker and Allan, 2012).

## Engineered Nanomedicines Targeted to BCSCs

**FIGURE 2:**
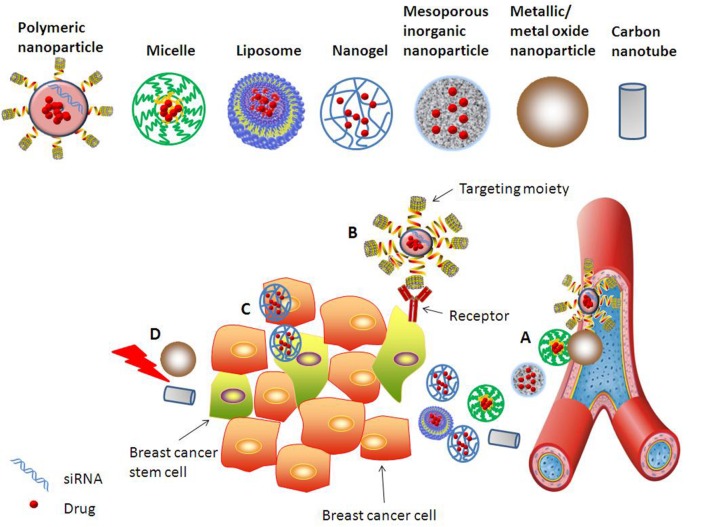
**Various approaches explored to target BCSCs using nanomedicines.** Different nanocarriers, such as polymeric nanoparticle, inorganic nanoparticle, micelle, liposome, nanogel, and nanotube, are developed for effective and specific drug/gene delivery to BCSCs. Strategies for improving anti-BCSCs therapeutic efficacy include but are not limited to: **(A)** Nanomedicines passively accumulating at the tumor site due to EPR effect. **(B)** Enhanced uptake of functionalized nanomedicines by BCSCs via receptor-mediated endocytosis. **(C)** Co-delivery of drugs targeting simultaneously BCSCs and bulk breast cancer cells. **(D)** Metallic or metal oxide nanoparticles and carbon nanotube mediated thermal therapy provides strategy for efficient inhibition of BCSCs.

Nanotechnology nowadays offers novel solutions in cancer therapy by enabling the engineered nanomedicines to navigate the body in very specific ways (Kievit and Zhang, 2011). Nanomedicines can solve the problems of drug solubility, instability, and short circulation half-life, and can co-deliver different drugs specifically to the target site. Due to enhanced permeability and retention (EPR) effect, nanotechnology-based drug delivery systems can passively accumulate at the tumor site. Modification of the nanocarriers surface with targeting moieties could generate enhanced specificity and cellular uptake in target cells (Zhao et al., 2013; Aires et al., 2016; Zuo et al., 2016). By careful control of sizes, components and targeting moieties, nanomedicines could be specifically targeted to BCSCs (**Figure 2**).

### Active Targeting Strategies for Anti BCSCs Therapy

Biological functionalization of the nanocarriers is promising strategy for improving selectivity of delivery systems toward specific cell types (Aires et al., 2016). In most cases, the delivery systems are equipped with certain agents that recognize and bind to the surface markers of BCSCs (**Table 1**).

**Table 1:** Targeting moieties for active BCSCs targeting.

Targeting moieties	Targets	Therapeutic agents	Delivery systems	Reference
Anti-CD44 antibody	CD44 receptor	Paclitaxel	(PLGA-co-PEG) polymeric micelles	Gener et al., 2015
Anti-CD44 antibody	CD44 receptor	Gemcitabine derivatives	Iron oxide magnetic nanoparticles	Aires et al., 2016
Hyaluronic acid	CD44 receptor	Etoposide/salinomycin/curcumin	Cholesteryl-hyaluronic acid nanogel-drug conjugates	Wei et al., 2013
Hyaluronic acid	CD44 receptor	Salinomycin	PLGA nanoparticles	Muntimadugu et al., 2016
Hyaluronic acid	CD44 receptor	8-hydroxyquinoline	Mesoporous silica nanoparticles-supported lipid bilayers	Wang et al., 2013
Hyaluronic acid	CD44 receptor	siRNA	Hyaluronic acid based versatile self-assembling nanosystems	Ganesh et al., 2013
Oligosaccharides of hyaluronan	CD44 receptor	Curcumin/paclitaxel	Inorganic calcium and phosphate ions coated hyaluronan oligosaccharides -histidine-menthone 1,2-glycerol ketal micelles	Chen et al., 2015
Chitosan	CD44 receptor	Doxorubicin	Pluronic F127-Chitosan nanoparticles	Rao et al., 2015
Anti-CD133 antibody	CD133 receptor	Paclitaxel	PLGA nanoparticles	Swaminathan et al., 2013
Vasoactive intestinal peptide	Vasoactive intestinal peptide receptors	Curcumin	Sterically stabilized phospholipid nanomicelles	Gülçür et al., 2013
Herceptin	Human epidermal growth factor receptor type 2	Salinomycin	PLGA nanoparticles	Aydın, 2013


The receptor CD44 is strongly expressed by the BCSCs and it is a signaling platform that integrates cellular microenvironmental cues with growth factor and cytokine signals (Lu et al., 2016), which often chosen to be the receptor for BCSCs targeting nanosystems. Gener et al. (2015) developed anti-CD44 antibodies functionalized and paclitaxel (PTX) loaded poly[(D,L-lactide-co-glycolide)-co-PEG] (PLGA-co-PEG) micelles, which effectively promoted the internalization of PTX in BCSCs. Aires et al. (2016) reported gemcitabine derivatives loaded multifunctionalized iron oxide magnetic nanoparticles (MNPs), which were modified with anti-CD44 antibody for CD44^+^ cancer cells specific treatment. The targeting of these nanoparticles to different CD44^+^ cancer cell lines including breast cancer stem-like cells was observed, and the potential of selective drug delivery was proven by the suppression of CD44^+^ cancer cells in pancreatic and breast cancers cell lines.

Hyaluronic acid (HA) has been reported as a ligand that can specifically bind to CD44 (Shen et al., 2016). Breast cancer cells are known to have greater uptake of HA than normal tissues, requiring HA for high P-gp expression, which contributes to multi-drug resistance (Götte and Yip, 2006). This makes HA an attractive targeting moiety in anti-BCSCs nanomedicines. Wei et al. (2013) developed cholesteryl-hyaluronic acid (CHA) nanogel-drug conjugates for efficient treatment of cancer cells and, especially, drug-resistant and CD44-expressing breast cancer stem-like cells. These nanogels were efficiently internalized via CD44 receptor-mediated endocytosis and interacted with the cancer cell membrane simultaneously. Functionalization with HA coating also improved the cellular uptake of salinomycin (SLM) nanoparticles by 1.5 folds (Muntimadugu et al., 2016). Wang et al. (2013) prepared a HA-mediated BCSCs targeting delivery system comprising of 8-hydroxyquinoline-loaded hyaluronan modified mesoporous silica nanoparticles (MSN)-supported lipid bilayers (HA-MSS). Their studies also showed that HA promoted the uptake of HA-MSS in BCSCs. Ganesh et al. (2013) developed a series of CD44-targeting HA-based self-assembling nanosystems for siRNA delivery and observed enhanced delivery efficiency and gene silencing activity of the siRNA in drug-resistant CD44 receptor-overexpressing tumor models. They found that several HA derivatives could transfect siRNAs into cancer cells that overexpressed the CD44 receptors. Free excess soluble HA could block the CD44 receptors, which consequently resulted in more than 90% inhibition of the cell uptake (Ganesh et al., 2013). Chen et al. (2015) prepared novel multifunctionalized hyaluronan oligosaccharides-histidine-menthone 1,2-glycerol ketal (oHM) conjugates for BCSCs targeting. The oHM conjugates, which were applied to form micelles, possess hyaluronan oligosaccharides as the target of CD44 receptor on BCSCs.

The chemical structure of chitosan partially resembles that of HA, except it is positively charged, which may make chitosan-modified nanoparticles bind more efficiently to mammalian cells. Therefore, Rao et al. (2015) constructed a kind of chitosan-decorated and doxorubicin-loaded polymeric nanoparticle that specifically targeted the CD44 receptors of BCSCs. This targeting nanoparticle increased the efficacy of doxorubicin for eliminating BCSCs by six times compared with free drug.

Choosing CD133 as the target receptor, Swaminathan et al. (2013) developed paclitaxel loaded and anti-CD133 antibody modified polymeric nanoparticles. Studies demonstrated that these nanoparticles efficiently eliminated BCSCs *in vitro* and significantly inhibited tumor-regrowth *in vivo*, which suggested CD133 might be a potential target for anti-BCSCs therapies.

Besides CD44 and CD133 receptors, some other targets could also be chosen for actively targeting the BCSCs. For example, there is a significantly higher level of vasoactive intestinal peptide (VIP) receptor expression in the BCSC population as compared to the bulk cancer cells, which makes the VIP a potential active targeting moiety to achieve high anti-BCSC activity (Gülçür et al., 2013). A novel sterically stabilized curcumin phospholipid nanomicelles (C-SSM) delivery platform surface conjugated with VIP has been found to successfully hinder the growth of breast cancer with BCSCs (Gülçür et al., 2013). Aydın (2013) applied Herceptin (HER) as the targeting ligand for salinomycin (SAL)-loaded PLGA nanoparticles in order to enhance the binding and accumulation of SAL in the breast cancer cells. HER is approved as an antibody for the treatment of human epidermal growth factor receptor type 2 (HER2)-positive metastatic breast cancers, and the receptor is overexpressed in 25–30% of invasive breast cancers. Since SAL can selectively deplete human BCSCs from tumorspheres and inhibit breast cancer growth, targeting SAL to the breast cancer bulk could lead to effective reduction of BCSCs.

### Nanoparticles for BCSCs Detection and Labeling

Although the BCSCs play critical roles in development and recurrence of breast cancer, they represent a minor subpopulation (2%) of the unfractionated breast cancer cells (Dick, 2003), making it difficult to isolate, detect and label them. Yet the detection and labeling of BCSCs is important in building a strong clinical foundation to further explore ways for the diagnosis and treatment of breast cancer at an early stage. Sung et al. (2009) constructed bifunctional cobalt ferrite magnetic nanoparticles coated with a silica shell. Rhodamine B isothiocyanate was incorporated into the silica shell, and the surface of the nanoparticles was modified with PEG. These nanoparticles could be efficiently internalized into stem cells, which then allow for the detection of the cells *in vitro* and *in vivo*, and the non-invasive demarcation of the cells by both optical imaging and magnetic resonance imaging (MRI). Park et al. (2014) described a multi-modal (MRI/optical) transfection agent (MTA) based on monodisperse magnetic nanoparticles for stem cell gene delivery and tracking. Stem cells after transplantation were successfully visualized via MRI and optical imaging over a 14-day period. Vuu et al. (2005) also fabricated a gadolinium-rhodamine nanoparticle for use in labeling and tracking cells via MRI and optical imaging. This nanoparticle could be modified with different fluorophores and targeting agents for studying its trafficking pathways in metastatic cells stem cells, and immune cells. These nanotechnology-based methods for CSCs labeling may possibly also be applied for detecting BCSCs in solid breast cancer tumors (Chen et al., 2016).

### Nanoparticles and Nanotubes for Mediating Thermal Therapy

Thermal therapy is an evolving and growing treatment option for breast cancer patients. Biologics and chemotherapeutics rely heavily on the inhibition of a particular molecular pathway, which can limit the number of responsive patients and give rise to the development of resistant cancer cells (Narayan et al., 2009; Korkaya et al., 2012; Paholak et al., 2016). In comparison, nanoparticle-mediated thermal therapy offers a broader applicability, including refractory subtypes such as triple negative breast cancer. Gold-based nanoparticles, iron oxide nanoparticles and carbon nanotubes have been the predominant platforms under investigation to produce targeted heat therapy against BCSCs in response to a near infrared (NIR) light source, or in the presence of an alternating magnetic field. (Atkinson et al., 2010; Burke et al., 2012; Sadhukha et al., 2013; Xu et al., 2014; Paholak et al., 2016).

Atkinson et al. (2010) applied intravenous injection gold nanoshells to target BCSCs and mediate hyperthermia. Using xenograft models of triple-negative breast cancer, they found that a subpopulation of cancer cells enriched in BCSCs was more resistant to ionizing radiation than the bulk tumor cells, and therefore the percentage of BCSCs increased after radiotreatment. In contrast, they used intravenously administered, optically activated gold nanoshells to generate local hyperthermia at 42°C for 20 min after ionizing radiation, and observed tumor size reduction without any significant increase in the relative proportion of BCSCs. Cells derived from the tumors treated with ionizing radiation plus hyperthermia exhibited both a marked decrease in tumorigenicity and a more differentiated phenotype than mock- and ionizing radiation–treated tumors, which suggested that hyperthermia sensitizes the BCSCs population to radiation treatment. Xu et al. (2014) found that gold nanorods (AuNRs)-mediated photothermal therapy (PTT) selectively eliminated BCSCs in MCF-7 breast cancer cells. It significantly reduced the ALDH^+^ cells subpopulation and the ability of the treated cells to form the mammosphere. Also, the gene expression of stem cell markers, including ALDH1 and KLF4, was decreased. Cellular uptake assay revealed that the polyelectrolyte-conjugated AuNRs could be more rapidly internalized by the BCSCs, and to a greater extent than non-cancer stem cells, which might be the main reason for the selective elimination of the BCSCs. There was also the potential to load salinomycin into the polyelectrolyte-conjugated AuNRs to obtain a synergistic effect on BCSCs inhibition via NIR light- triggered drug release and hyperthermia therapy.

Biodegradable and highly crystallized iron oxide nanoparticles (HCIONPs) were also applied to mediate PTT for effectively eliminating BCSCs in translational models of triple negative breast cancer (Paholak et al., 2016). The intravenously injected HCIONPs (∼15 nm diameter) effectively accumulated in the breast cancer tumors via the EPR effect, and their highly crystallized structure enabled effective tumor heating upon stimulation by NIR light. PTT applied *in vitro* preferentially targeted the epithelial-like ALDH^+^ BCSCs, followed by mesenchymal-like CD44^+^/CD24^-^ BCSCs, compared to bulk cancer cells. The PTT inhibited the capacity of the BCSC for self-renewal through reduction of mammosphere formation in primary and secondary generations. Implantation of HCIONPs in NOD/SCID mice allowed the PTT to effectively impede BCSC-driven tumor formation. Further studies on the translational potential of PTT using metastatic and immune-competent mouse models showed that the PTT could inhibit BCSCs significantly, and reduced metastasis to the lung and lymph nodes.

In another study, superparamagnetic iron oxide nanoparticles (SPIO NPs) with a magnetite core of 12 nm were applied to induce magnetic hyperthermia for the selective CSCs inhibition (Sadhukha et al., 2013). SPIO NPs were placed in an alternating magnetic field to generate localized heat in lung tumor cells A549 and breast tumor cells MDA-MB-231. Multiple assays *in vitro* and *in vivo* revealed that magnetic hyperthermia markedly reduced or eliminated the BCSC subpopulation. In contrast, conventional hyperthermia in water bath had not similar effect.

Carbon nanotubes (CNTs) are also candidate platforms for mediating thermal therapy against breast cancer. Using a model of triple-negative BCSCs, Burke et al. (2012) showed that BCSCs lost their long-term proliferative capacity after nanotube-mediated thermal therapy. In contrast, the BCSCs were resistant to conventional hyperthermia and a concomitant increase in the percentage of these cells was observed. Moreover, use of nanotube-mediated thermal therapy *in vivo* promoted complete regression of tumor and long-term survival of mice bearing cancer stem cell-driven breast tumors. Mechanistically, the nanotube-mediated thermal therapy promoted rapid membrane permeabilization and necrosis of BCSCs. It was suggested that the nanotube-mediated thermal treatment could simultaneously eliminate both the differentiated cells and the BCSCs that drove tumor growth and recurrence.

### Nanocarriers for BCSCs-Targeted Drug Delivery

Various kinds of nanocarriers, including polymeric nanoparticles, inorganic nanoparticles, liposomes, micelles, and nanogels, have been explored in recent years for BCSCs-targeted drug delivery. These delivery platforms effectively improved drug stability and enabled the controlled release of high concentrations of multicomponent cargos to breast tumor cells and/or BCSCs. Here, we summarize and categorize the reported nanomedicines which have been introduced and evaluated for BCSCs-targeted drug delivery (**Table 2**).

**Table 2:** Nanocarriers for BCSCs-targeted drug delivery.

Delivery systems	Therapeutic agents	Targeting moieties	Mechanisms of action	Reference
PEG-b-PLA nanoparticles	Bortezomib		Increased drug accumulation within cancer cells	Shen et al., 2015
PEG-b-PLA nanoparticles	Decitabine/doxorubicin		Combination of drugs for sensitizing BCSCs to chemotherapy	Li et al., 2015
PEG-b-PLA nanoparticles	All-*trans*-retinoic acid/doxorubicin		Co-delivery of drugs for inducing BCSCs to differentiate into non-CSCs and inhibiting tumor cell	Sun et al., 2015
PEG-b-PLA nanoparticles	Chloroquine/doxorubicin/docetaxel		Co-delivery of drugs for sensitizing BCSCs to chemotherapy	Sun et al., 2016
PLGA nanoparticles	Salinomycin/paclitaxel	Hyaluronic acid	Co-delivery of drugs targeted toward BCSCs and bulk breast cancer cells	Muntimadugu et al., 2016
PLGA nanoparticles	Paclitaxel	Anti-CD133 monoclonal antibody	Selective inhibition of BCSCs	Swaminathan et al., 2013
iTEP nanoparticles	Salinomycin		Increased drug accumulation within cancer cells	Zhao et al., 2014
Chitosan modified Pluronic F127 nanoparticles	Doxorubicin	Chitosan	Selective inhibition of BCSCs	Rao et al., 2015
Cationic lipid-assisted polymeric nanoparticles	siRNA		Increased accumulation of siRNA within cancer cells	Zuo et al., 2016
HA based self-assembled nanosystems	siRNA	Hyaluronic acid	Increased accumulation of siRNA within cancer cells	Ganesh et al., 2013
Gold nanoparticles	Doxorubicin		Increased accumulation of drug within BCSCs	Sun et al., 2014
Iron oxide magnetic nanoparticles	Gemcitabine derivatives	Anti-CD44 antibody	Selective inhibition of BCSCs	Aires et al., 2016
Hyaluronan modified/non-modified mesoporous silica nanoparticles -supported lipid bilayers	8-hydroxyquinoline/docetaxel	Hyaluronan	Combination therapy	Wang et al., 2013
Zinc sulfide nanoparticles			Selective inhibition of BCSCs	Tran et al., 2016
PEG-b-PCL micelles	Paclitaxel/salinomycin	Octreotide	Combination therapy	Zhang et al., 2012
PLGA-co-PEG micelles	Paclitaxel	Anti-CD44 antibody	Selective inhibition of BCSCs	Gener et al., 2015
PEG-PAC and PEG-PUC mixture micelles	Thioridazine/doxorubicin		Co-delivery of drugs targeted toward BCSCs and bulk breast cancer cells	Ke et al., 2014
Phospholipid nanomicelles	Curcumin	Vasoactive intestinal peptide	Selective inhibition of BCSCs	Gülçür et al., 2013
Inorganic calcium and phosphate ions coated micelles	Curcumin/paclitaxel	Hyaluronan oligosaccharides	Co-delivery of drugs targeted toward BCSCs	Chen et al., 2015
Cross-linked multilamellar liposomal vesicles	Salinomycin/doxorubicin		Co-delivery of drugs targeted toward BCSCs and bulk breast cancer cells	Kim et al., 2015
Liposomes	Dexamethasone/ESC8/NRP-1 shRNA-encoded plasmid		Co-delivery of drugs and shRNA-encoded plasmid	Ahmad et al., 2016
Stealth liposomes	All-*trans* retinoic acid/vinorelbine		Prolonged circulation, improved biodistribution and accumulation of drugs within tumors	Li et al., 2011
Stealth liposomes	Vinorelbine/parthenolide		Prolonged circulation, improved biodistribution and accumulation of drugs within cancer cells	Liu et al., 2008
Nanogels	Etoposide/salinomycin/curcumin	Hyaluronic acid	Increased accumulation of drug within cancer cells	Wei et al., 2013


#### Polymeric Nanoparticles

Polymeric nanoparticles (PNPs) are submicron-sized colloidal particles, and are believed to be the simplest nanomedicine form of soft-materials (Bobo et al., 2016). An anticancer agent is adsorbed, encapsulated or conjugated either within or onto the surface of the PNPs, which are capable of providing a required dose of the anticancer agent to the tumor site for a sustained period of time. PNPs with labile surface moieties could be readily modified and functionalized to impart desirable drug delivery properties, thereby providing a valuable approach to develop “magic bullet” strategies to deliver one or more therapeutic agents to BCSCs (Masood, 2016).

The bortezomib (BTZ) loaded poly(ethylene glycol)-block-poly(D,L-lactide) (PEG-b-PLA) nanoparticles (NP_BTZ_) was prepared to enhance the water solubility and stability of BTZ and to promote anticancer effect (Shen et al., 2015). NP_BTZ_ effectively delivered BTZ into both BCSCs and non-CSCs, resulting in proliferation inhibition and initiation of apoptosis. Delivery via the nanoparticles increased BTZ uptake into both adherent cells and mammosphere cells and adversely affected the ‘stemness’ of BCSCs compared with free BTZ. Prolonged circulation half-life of BTZ and enhanced drug accumulation within the tumor tissue were also observed. Li et al. (2015) encapsulated decitabine (DAC) in PEG-b-PLA nanoparticles to sensitize both BCSCs and non-CSCs to the chemotherapy. According to the *in vitro* data, treatment with DAC loaded nanoparticles (NP_DAC_) combined with doxorubicin loaded nanoparticles (NP_DOX_) better reduced the percentage of BCSCs in the mammospheres of MDA-MB-231 cells and overcame the drug resistance more effectively. The expression of DNMT1 and DNMT3b was revealed to be significantly downregulated with the treatment of NP_DAC_ in a MB-MDA-231 xenograft model and the expression of caspase-9 was induced to increase, which contributed to the increased susceptibility of BCSCs and bulk cancer cells to NP_DOX_. The combination treatment of NP_DAC_ and NP_DOX_ resulted in the decreased proportion of ALDH^hi^ BCSCs, increased proportion of apoptotic tumor cells, and the suppression of breast cancer cell growth. Sun et al. (2015) encapsulated doxorubicin (DOX) and all-trans-retinoic acid (ATRA) in the same PEG-b-PLA nanoparticles on the basis that ATRA could induce the differentiation of BCSCs, which then reduced their tumor initiating ability and increased their sensitivity to DOX. The encapsulated ATRA and DOX both presented with slower *in vitro* release profiles compared to free drugs. Compared to NP_DOX_, NP_ATRA_ and NP_ATRA_-NP_DOX_ mixture, the NP_ATRA/DOX_ co-delivery systems was superior, both *in vitro* and *in vivo*, in suppressing tumor growth. Post-treatment, the BCSCs were induced to differentiate into non-CSCs and tumor cell growth was inhibited without triggering BCSCs enrichment. The prolonged circulation half-lives, improved pharmacokinetics and increased tumor uptake were added advantages of this dual drug-loaded nanoparticles system. The same group (Sun et al., 2016) then investigated the role of autophagy inhibition in influencing the susceptibility of BCSCs to chemotherapeutics. Chloroquine (CQ)-mediated autophagy inhibition was showed to reduce ‘stemness’ and increase sensitivity of sorted ALDH^hi^ cells to chemotherapeutics (DOX and docetaxel, DTXL) in breast cancer cell MDA-MB-231, and the effects were further promoted by the PEG-b-PLA nanoparticle-based delivery system *in vitro*. Prolonged circulation half-life and enhanced accumulation of the drugs within the tumor tissues and ALDH^hi^ cells were observed *in vivo*. The combined NP_CQ_/NP_DOX_ (NP_CQ_ and NP_DOX_ mixture) and NP_CQ/DOX_ (NP_CQ/DOX_ co-delivery systems) treatments showed augmented tumor growth inhibitory effect by killing both the BCSCs and bulk tumor cells in the MDA-MB-231 orthotopic tumor murine model.

Poly(lactic-co-glycolic acid) (PLGA) nanoparticles were designed for co-delivering SAL and PTX to breast cancer cells with SAL targeted toward BCSCs whereas PTX was used to kill all cancer cells (Muntimadugu et al., 2016). Active targeting of BCSCs was achieved through incorporation of HA onto the surface of the SAL-loaded nanoparticles. *In vitro* cytotoxicity studies revealed that combination of HA-coated SAL nanoparticles and PTX nanoparticles were more potent than all other formulations. The synergistic action of SAL and PTX, and their specificity toward the targeted cells, resulted in high cytotoxicity against the CD44^+^ breast cancer stem-like cells. In another study, PTX-loaded PLGA nanocarriers were conjugated with an anti-CD133 monoclonal antibody to target the CD133 marker on BCSCs (Swaminathan et al., 2013). *In vitro* experiments showed that the PTX-loaded CD133NPs effectively decreased the number of mammospheres and colonies formed, while free PTX could not eliminated BCSCs efficiently. In the MDA-MB-231 xenograft model, free PTX treatment induced tumor regrowth, which was avoided when PTX CD133NPs were utilized, suggesting that the encapsulation of PTX in CD133NPs could significantly decrease the BCSCs and improve therapeutic efficacy.

A kind of hydrophilic, immune-tolerant, elastin-like polypeptide (iTEP) was used to develop nanoparticles for the delivery of SAL to BCSCs in breast tumors (Zhao et al., 2014). To construct this nanoparticle system, hydrophobic SAL was conjugated to iTEP; the amphiphilic iTEP-SAL conjugates self-assemble into nanoparticles, and free SAL was encapsulated into the nanoparticles with two additives, N,N-dimethylhexylamine (DMHA) and α-tocopherol. The dual-encapsulation technique significantly improved the drug loading efficiency and release profile, plasma drug concentration-time area under curve and tumor accumulation of SAL, which led to a boost of BCSCs-elimination effect, which translated to a delay in tumor growth *in vivo*. Post-treated 4T1 orthotopic tumors showed a mean BCSCs frequency of 55.62%, a significant reduction from the mean frequencies of 75.00% for untreated tumors, and 64.32% for tumors treated with free SAL.

Rao et al. (2015) developed a DOX-encapsulated polymeric nanoparticle synthesized using Pluronic F127 cross-linked and surface-decorated with chitosan that can specifically target the CD44 receptors of cancer cells. This nanoparticle system was engineered to release the DOX in acidic environments, which occurs when the nanoparticles were localized in the acidic tumor microenvironment and in the cellular endosomes/lysosomes. This delivery system increased the efficacy of DOX by six times for eliminating CD44^+^ breast cancer stem-like cells compared with free DOX. In orthotopic xenograft tumor model, these nanoparticles also reduced the size of tumors efficiently.

#### Inorganic Nanoparticles

Inorganic nanoparticles include a large number of inorganic platforms (e.g., metallic, metal oxide and silica nanoparticles), which could be applied as the drug carriers or as the therapeutic agents (Bobo et al., 2016).

Sun et al. (2014) developed DOX-tethered gold nanoparticles to mediate DOX delivery to BCSCs. This system was found to be highly potent, reducing the BCSC mammosphere formation and cancer initiation activities, and eliciting marked enhancement in tumor growth inhibition in murine models. By evading the P-gp efflux pump, these DOX-Hyd@AuNPs nanoparticles were able to overcome the intrinsic resistance to deliver more DOX into the BCSCs. The nanoparticles also markedly attenuated tumor growth during the off-therapy stage by reducing the number of BCSCs in tumors, in contrast with the therapy with DOX alone, which evoked an enrichment of BCSCs.

Gemcitabine derivatives loaded iron oxide magnetic nanoparticles with anti-CD44 antibody modification were constructed for the selective treatment of CD44-positive cancer cells (Aires et al., 2016). This nanoparticle delivery system was able to target different CD44-positive cancer cell lines, and to selectively kill CD44-positive cancer cells present in pancreatic and breast cancer cell lines.

Wang et al. (2013) developed 8-hydroxyquinoline (8-HQ)-loaded hyaluronan modified MSN-supported lipid bilayers (HA-MSS) and DTXL-loaded MSS to achieve the goal of both targeting BCSCs and bulk breast cancer cells. It was demonstrated that HA promoted the uptake of HA-MSS in CD44-overexpressing MCF-7 mammospheres consisting of bulk cells and BCSCs, revealing the mechanism of receptor-mediated endocytosis. DTXL or DTXL-loaded MSS showed much enhanced cytotoxicity against MCF-7 cells than against MCF-7 mammospheres, whereas 8-HQ or 8-HQ-loaded HA-MSS showed much enhanced cytotoxicity against MCF-7 BCSCs compared with MCF-7 cells. When applied against the MCF-7 xenografts in mice, the combination therapy with DTXL-loaded MSS plus 8-HQ-loaded HA-MSS produced the strongest antitumor efficacy, with little systemic toxicity in mice.

Zinc sulfide (ZnS) nanoparticles were reported to possess inhibitory effects toward the migration and invasion of BCSCs in MCF-7-SC with no apparent cytotoxicity up to a concentration of 400 μg/mL (Tran et al., 2016). The ZnS nanoparticles significantly inhibited the wound healing in the MCF-7-SC cells. The results suggested that ZnS nanoparticles inhibited the metastasis of MCF-7-SCs in a dose-dependent manner by suppressing the epithelial-mesenchymal transition process.

#### Polymeric Micelles

Polymeric micelles are derived from self-assembled amphiphilic block copolymers. Therefore these colloidal particles contain both hydrophilic and hydrophobic components, which provide platforms for various modifications to improve targeting efficiency. Polymeric micelles have been popular drug carriers for anticancer treatment due to their uniformity, small sizes, and prolonged circulation time (Torchilin, 2007; Jeong et al., 2016).

Zhang et al. (2012) prepared PEG-b-PCL polymeric micelles modified with octreotide (Oct) and loaded with PTX (Oct-M-PTX) as well as PEG-b-PCL polymeric micelles loaded with SAL (M-SAL) using the thin film hydration method. Oct was coupled to the PEG end of PEG-b-PCL before micelles preparing for the specific targeting toward somatostatin receptors (SSTR) overexpressed in breast cancer. It was observed that the uptake of Oct modified micelles was significantly promoted in SSTR overexpressed MCF-7 cells while the promotion could be inhibited by free Oct, confirming the receptor-mediated endocytosis mechanism. M-SAL was capable of decreasing the proportion of BCSCs. Oct-M-PTX exhibited stronger inhibitory action against the MCF-7 cells compared with PTX-loaded micelles (M-PTX), and synergistic effect was found when Oct-M-PTX and M-SAL were utilized in combination. Similar synergistic effect was also observed in the MCF-7 xenografts. *In vivo* studies revealed that M-SAL was more effective in inhibiting BCSCs compared with free SAL. In another study, PTX loaded and anti-CD44 antibodies functionalized PLGA-co-PEG polymeric micelles were prepared for treatment against breast cancer cell lines (Gener et al., 2015). The results showed that the encapsulation of PTX into the targeted PLGA-co-PEG micelles increased sensitivity of BCSCs to PTX. Ke et al. (2014) utilized a mixture of acid-functionalized poly(carbonate) and poly(ethylene glycol) diblock copolymer (PEG-PAC) and urea-functionalized poly(carbonate) (PUC) and PEG diblock copolymer (PEG-PUC) to form micelles via self-assembly. These mixed micelles (MM) were applied to co-deliver thioridazine (THZ) and DOX targeting both breast cancer cells and BCSCs. THZ and THZ-MM were effective in inhibiting BCSCs, while DOX was efficient in suppressing non-stem-like cancer cells. Co-delivery of free THZ and DOX or THZ-MM and DOX-MM resulted in a stronger inhibitory effect on BCSCs, as compared with free DOX or DOX-MM alone. THZ and THZ-MM were capable of lowering the population of BCSCs in unsorted cells. In the BT-474 xenografts, the co-delivery of DOX-MM and THZ-MM produced the strongest antitumor efficacy, and both THZ and THZ-MM showed strong activity against BCSCs. Gülçür et al. (2013) investigated the potential of using curcumin (CUR)-loaded sterically stabilized phospholipid nanomicelles (C-SSM) surface conjugated with VIP (C-SSM-VIP) to target and hinder breast cancer with BCSCs. The *in vitro* experiments revealed that the nanomicellar C-SSM-VIP resulted in up to 20% inhibition of tumorsphere formation at a dose of 5 μM of curcumin.

More complex polymeric micellar delivery systems have been constructed. For example, a multifunctional oligosaccharides of hyaluronan (oHA) conjugates, oHA-histidine-menthone 1,2-glycerol ketal (oHM) was prepared to form micelles carriers for BCSCs targeting (Chen et al., 2015). The oHM conjugates possessed the oHA to target the CD44 receptor, and these conjugates self-assembled into micelles. CUR and PTX were loaded into the oHM micelles. Novel “nano-eggs” were then fabricated through controlled deposition of inorganic calcium and phosphate ions onto the surface of the micelles via a sequential addition method. The size of nano-eggs (120.6 ± 4.5 nm) was smaller than that of oHM micelles (158.6 ± 6.4 nm), indicating that the micelles might be compacted by mineralization. The “nano-eggs” were stable at pH 7.4, while in acidic conditions (pH 6.5), the inorganic minerals outer shells were destroyed. The loaded CUR and PTX were released in a sustained manner from both the nano-eggs and oHM micelles depending on the solution pH. *In vivo*, the nano-eggs showed stronger tumor targeting effects than the oHM micelles. The antitumor efficacy against MDA-MB-231 xenografts in nude mice revealed that the co-delivery of PTX and CUR via the nano-eggs produced the strongest antitumor efficacy, with the nano-eggs showing strong activity against the BCSCs.

#### Liposomes

Liposomes are colloidal nanocarriers consisting of amphiphilic phospholipid bilayers, which can be loaded with both hydrophilic and hydrophobic drugs. Liposomes are ideal nanocarriers for anti-BCSCs therapies owing to their desirable characteristics such as biocompatibility, ease of surface modification, and long blood circulation time (Jeong et al., 2016).

All-*trans*-retinoic acid (ATRA) stealth liposomes have been studied for their ability to prevent the relapse of breast cancer arising from BCSCs, and as co-therapy with a cytotoxic agent for treating breast cancer (Li et al., 2011). Stealth property was imparted to the liposomes by modification with PEG derivative. Efficacies of action were confirmed *in vitro* and in t BCSCs xenografts in NOD/SCID mice. The ATRA stealth liposomes were shown to prevent the relapse of breast cancer by promoting BCSCs differentiation and arresting the cell-cycle. They could also be used to treat breast cancer in combination with vinorelbine stealth liposomes (Li et al., 2011). In another study, vinorelbine stealth liposomes and parthenolide stealth liposomes given as a combination were shown to provide synergistic pharmacological properties to inhibit the proliferation of BCSCs and non-stem cancer cells in the MCF-7 and MDA-MB-231 cell models (Liu et al., 2008). A more robust inhibitory effect on the BCSCs was observed with the combination treatment than treatment with vinorelbine alone. In the MCF-7 xenografts, the combination treatment produced a full inhibitory effect.

#### Nanogels

Nanogels, which are hydrogel materials confined to nanoscopic dimensions, are being explored as drug delivery agents for targeting cancer due to their easy tailoring properties. They can also efficiently encapsulate diverse therapeutics through simple fabrication mechanisms (Soni and Yadav, 2016).

Based on cholesteryl-HA (CHA), nanogel-drug conjugates were synthesized to target and inhibit drug-resistant tumors (Wei et al., 2013). Some poorly soluble drugs with activity against CSCs, such as SAL, CUR, and etoposide (ETO), had undesirable bioavailability, which could be significantly ameliorated by the conjugates. Small nanogel particles were formed by ultrasonication. Following the hydrolysis of biodegradable ester linkages, drugs were released from nanogels in a sustained manner. The suppression of CD44-expressing drug-resistant human breast and pancreatic adenocarcinoma cells by CHA-drug nanogels was 2–7 times more effective than that by free drugs or non-modified HA-drug conjugates. Via CD44 receptor-mediated endocytosis, the nanogels were internalized efficiently and interacted with the cancer cell membrane simultaneously. CHA-drug nanogels could be anchored by cholesterol moieties in the cellular membrane after unfolding, which evidently resulted in more drug accumulation in the cancer cells compared with HA-drug conjugates without cholesteryl modification. CHA-drug nanogels penetrated multicellular cancer spheroids more efficiently and displayed stronger inhibitory effects in the system modeling tumor environment than both HA-drug conjugates and free drugs.

#### Nanocarriers for siRNA Delivery

RNA interference (RNAi) has emerged as a powerful strategy for BCSCs therapy by selective down-regulation of key genes. However, the delivery of small interfering RNAs (siRNAs) to specific tumor tissues and cells is a major hurdle that remains to be addressed before this experimental technique can become a clinically viable therapeutic option (Ganesh et al., 2013). Systemic siRNA delivery systems should have properties that include biocompatibility, biodegradability, and non-immunogenicity. Additionally, the systems should protect the siRNA load from serum nucleases, and provide effective targeted delivery that includes a mechanism for siRNA endosomal escape to enter the RNAi machinery and activate RNAi pathways (Xu and Wang, 2015).

To develop an effective siRNA delivery system for anti-BCSCs therapy, Zuo et al. (2016) constructed cationic lipid-assisted polymeric nanoparticles encapsulated with siRNA via a double emulsion method. The nanoparticles loaded with siRNA targeting the oncogene polo-like kinase 1 (Plk1) effectively eliminated the BCSCs *in vitro*. However, the BCSCs in the residual tumor tissue became enriched following systemic treatment. LY364947, an inhibitor of TGF-b type I receptor, could inhibit the TGF-b signaling pathway, and therefore promoted the penetration of nanoparticles in the tumor tissue. With the help of TGF-b signaling pathway inhibition, the intratumoral distribution of nanoparticles in the MDA-MB-231 xenografts was significantly ameliorated and the therapeutic siRNA was successfully delivered to the BCSCs *in vivo*. LY364947 altered the tumor microenvironment and improved the EPR effect, thereby ultimately improved *in vivo* transfection efficiency of BCSCs.

Ganesh et al. (2013) prepared and screened a series of HA based self-assembling nanosystems for targeted delivery of siRNA. Polyamines and lipids of varying carbon chain lengths/nitrogen content were utilized to functionalize the HA polymers. Nanosystems derived from many HA derivatives transfected siRNA efficiently into CD44 overexpressing cancer cells including MDA-MB468 breast cancer cells. These siRNA encapsulated nanosystems selectively accumulated at tumor site and targeted specific gene knock down in both solid tumors and metastatic tumors *in vivo*. These HA based siRNA delivery systems thus portend to be promising for systemic targeted therapeutics against CD44 overexpressing breast cancer stem-like cells and metastatic lesions.

## Conclusion and Prospects

Nanomedicines seem to be efficient candidates for BCSCs therapy. Multifunctional nanomedicines offer a range of strategies for targeting one or more therapeutics to BCSCs, increasing their cellular uptake, prolonging systemic circulation, improving biodistribution profiles, and resolving problems of poor stability and solubility. However, the application of technology-based anti-BCSCs therapeutics is in a relatively early stage. To advance these nanotechnologies for anti-BCSCs clinical therapy, more efforts are needed. Firstly, a better understanding of BCSCs microenvironment biology and key characteristics is necessary for the fabrication of more effective anti-BCSCs delivery systems. Additional genetic/molecular markers that are more specific to BCSCs may have to be identified to optimize the active BCSCs targeting strategies. Intelligent delivery systems could be designed that incorporate a BCSCs niche sensitive triggered drug release strategy. Secondly, mechanisms involved in the epigenetic regulation of BCSC self-renewal and non-BCSC reprogramming needs to be thoroughly understood. Based on these understandings, more effective chemo- or gene therapies, as well as better delivery systems, are likely to be designed and constructed. Thirdly, comprehensive therapeutic systems affecting both BCSCs and bulk breast cancer cells are needed. A successful therapy will encompass the prevention of breast cancer initiation, progression, resistance, recurrence, and metastasis. According to the CSCs theory, BCSCs and differentiated breast cancer cells may transform into each other via unknown pathway(s). Therapies that kill only the BCSCs or breast cancer cells could possibly cause recurrence. Synergistic delivery systems that eliminate BCSCs and non-BCSCs simultaneously will be preferred. Aside from BCSC biology, the unsatisfactory stability of nanomedicines and associated potential for leakage of loaded drugs during blood circulation are concerns that are yet to be resolved. Strategies such as surface modification or coating are needed, and more basic and applied researches *in vivo* are necessary. In summary, nanotechnology based BCSCs targeting treatment provides an attractive strategy for therapy of breast cancers. Although a concerted effort is required to tackle the challenges outlined above, there is great optimism that the near future will see successful clinical translation of BCSCs-targeted nanomedicines to provide unique benefit for cancer patients.

## Author Contributions

LH and JG summarized the literatures and wrote the manuscript. LL revised and edited the manuscript. Z-xY provide critical comments and revised the manuscript. JM revised the manuscript and supervised all the works.

## Conflict of Interest Statement

The authors declare that the research was conducted in the absence of any commercial or financial relationships that could be construed as a potential conflict of interest.
